# Supplementary material for: Characteristics of a Danish Post-COVID Cohort Referred for Examination due to Persistent Symptoms Six Months after Mild Acute COVID-19
Source: J Clin Med. 2022 Dec 10;11(24):7338. doi: 10.3390/jcm11247338 (PMC9783804; doi:10.3390/jcm11247338)
Supplement: Supplementary file 1 [file jcm-11-07338-s001.zip › Table S1.pdf]

MULTICOV Study Group

Name	Affiliation and titel	ORCID
Anders Lehmann Dahl Peder	1. Department of Cardiology, Aarhus University Hospital, Palle Juul-Jensens Boulevard 99, 8200 Aarhus, Denmark:<br>2. Department of Clinical Medicine, Aarhus University, Palle Juul-Jensens Blvd. 82, 8200 Aarhus N, Denmark:	0000-0002-3258-5192
Andreas Fløe Hvass	1. Department of Respiratory Diseases and Allergy, Aarhus University Hospital, Palle Juul-Jensens Boulevard 99, 8200 Aarhus, Denmark:<br>2. Department of Clinical Medicine, Aarhus University, Palle Juul-Jensens Blvd. 82, 8200 Aarhus N, Denmark:	0000-0003-2787-0041
Benjamin Yamin Ali Khan	1. Department of Neurology, Aarhus University Hospital, Palle Juul-Jensens Boulevard 99, 8200 Aarhus, Denmark:<br>2. Department of Clinical Medicine, Aarhus University, Palle Juul-Jensens Blvd. 82, 8200 Aarhus N, Denmark: phd-student	
Berit Schiøttz-Christensen	1. Department of Infectious Diseases, Aarhus University Hospital, Palle Juul-Jensens Boulevard 99, 8200 Aarhus, Denmark: staff specialist.<br>2. Research Unit of General Practice, Department of Public Health, University og Southern Denmark, J.B. Winsløws Vej 9B, 2nd floor, Odense C - DK-5000: professor.	0000-0003-3866-856
Cagla Cömert	1. Research Unit for Molecular Medicine (MMF), Department of Clinical Medicine, Aarhus University, Palle Juul-Jensens Blvd. 82, 8200 Aarhus N, Denmark:<br>2. Department of Clinical Biochemistry, Aarhus University Hospital, Palle Juul-Jensens Boulevard 99, 8200 Aarhus, Denmark: post.doc	0000-0001-9905-3644
Christoffer Laustsen	1. Department of Radiology, Aarhus University Hospital, Palle Juul-Jensens Boulevard 99, 8200 Aarhus, Denmark: senior consultant.<br>2. The MR Research Centre, Department of Clinical Medicine, Aarhus University, Palle Juul-Jensens Blvd. 82, 8200 Aarhus N, Denmark: professor.	0000-0002-0317-2911
Elisabeth Bendstrup	1. Department of Respiratory Diseases and Allergy, Aarhus University Hospital, Palle Juul-Jensens Boulevard 99, 8200 Aarhus, Denmark: senior consultant.<br>2. Department of Clinical Medicine, Aarhus University, Palle Juul-Jensens Blvd. 82, 8200 Aarhus N, Denmark: professor.	0000-0002-4238-6963

Gregory Wood	1. Department of Cardiology, Aarhus University Hospital, Palle Juul-Jensens Boulevard 99, 8200 Aarhus, Denmark:<br>2. Department of Clinical Medicine, Aarhus University, Palle Juul-Jensens Blvd. 82, 8200 Aarhus N, Denmark: phd-student	
Hans Erik Bøtker	Faculty of Health, Aarhus University, Vennelyst Boulevard 4, DK-8000 Aarhus: Vice Dean for Research, professor.	0000-0001-6358-8962
Hatice Tankisi	1. Department of Neurology, Aarhus University Hospital, Palle Juul-Jensens Boulevard 99, 8200 Aarhus, Denmark: senior consultant.<br>2. Department of Clinical Medicine, Aarhus University, Palle Juul-Jensens Blvd. 82, 8200 Aarhus N, Denmark: professor.	0000-0001-8495-9769
Henning Andersen	1. Department of Neurology, Aarhus University Hospital, Palle Juul-Jensens Boulevard 99, 8200 Aarhus, Denmark: senior consultant.<br>2. Department of Clinical Medicine, Aarhus University, Palle Juul-Jensens Blvd. 82, 8200 Aarhus N, Denmark: professor.	0000-0002-9082-2694
Jane Agergaard	1. Department of Infectious Diseases, Aarhus University Hospital, Palle Juul-Jensens Boulevard 99, 8200 Aarhus, Denmark: staff specialist.<br>2. Department of Clinical Medicine, Aarhus University, Palle Juul-Jensens Blvd. 82, 8200 Aarhus N, Denmark: Associate professor.	
Johan Palmfeldt	1. Research Unit for Molecular Medicine (MMF), Department of Clinical Medicine, Aarhus University, Palle Juul-Jensens Blvd. 82, 8200 Aarhus N, Denmark: associate professor.<br>2. Department of Clinical Biochemistry, Aarhus University Hospital, Palle Juul-Jensens Boulevard 99, 8200 Aarhus, Denmark	0000-0001-5585-639X
Kristoffer Skaalum	1. Department of Infectious Diseases, Aarhus University Hospital, Palle Juul-Jensens Boulevard 99, 8200 Aarhus, Denmark:<br>2. Department of Clinical Medicine, Aarhus University, Palle Juul-Jensens Blvd. 82, 8200 Aarhus N, Denmark: phd-student	0000-0002-0902-352X
Lars Jørgen Østergaard	1. Department of Infectious Diseases, Aarhus University Hospital, Palle Juul-Jensens Boulevard 99, 8200 Aarhus, Denmark: chief physician.<br>2. Department of Clinical Medicine, Aarhus University, Palle Juul-Jensens Blvd. 82, 8200 Aarhus N, Denmark: professor.	0000-0003-2459-0511

Line Vibholm	1. Department of Cardiology, Aarhus University Hospital, Palle Juul-Jensens Boulevard 99, 8200 Aarhus, Denmark:<br>2. Department of Clinical Medicine, Aarhus University, Palle Juul-Jensens Blvd. 82, 8200 Aarhus N, Denmark:	0000-0001-8945-1905
Martin Mølhave	1. Department of Infectious Diseases, Aarhus University Hospital, Palle Juul-Jensens Boulevard 99, 8200 Aarhus, Denmark:<br>2. Department of Clinical Medicine, Aarhus University, Palle Juul-Jensens Blvd. 82, 8200 Aarhus N, Denmark:	
Rikke Katrine Jentoft Olsen	1. Research Unit for Molecular Medicine (MMF), Department of Clinical Medicine, Aarhus University, Palle Juul-Jensens Blvd. 82, 8200 Aarhus N, Denmark: associate professor.<br>2. Department of Clinical Biochemistry, Aarhus University Hospital, Palle Juul-Jensens Boulevard 99, 8200 Aarhus, Denmark.	0000-0003-3470-5589
Sofie Eg Jørgensen	1. Department of Infectious Diseases, Aarhus University Hospital, Palle Juul-Jensens Boulevard 99, 8200 Aarhus, Denmark:<br>2. Department of Biomedicine, Aarhus University, Hoegh Guldbergs gade 10, 8000 Aarhus C, Denmark: post.doc	0000-0002-2700-0882
Steen Hvitfeldt Poulsen	1. Department of Cardiology, Aarhus University Hospital, Palle Juul-Jensens Boulevard 99, 8200 Aarhus, Denmark: senior consultant.<br>2. Department of Clinical Medicine, Aarhus University, Palle Juul-Jensens Blvd. 82, 8200 Aarhus N, Denmark: professor.	0000-0002-9586-835X
Steffen Leth	1. Dep. of Infectious Diseases, Dep. of Internal Medicine, Gødstrup Hospital, Hospitalsparken 15, 7400-Herning, Denmark: Senior Registrar.<br>2. Department of Clinical Medicine, Aarhus University, Palle Juul-Jensens Blvd. 82, 8200 Aarhus N, Denmark: Clinical associate professor.	0000-0003-3194-9844
Søren Sperling Haugen	1. Department of Respiratory Diseases and Allergy, Aarhus University Hospital, Palle Juul-Jensens Boulevard 99, 8200 Aarhus, Denmark:<br>2. Department of Clinical Medicine, Aarhus University, Palle Juul-Jensens Blvd. 82, 8200 Aarhus N, Denmark: phd-student	0000-0001-5700-3948
Thomas Harboe	1. Department of Neurology, Aarhus University Hospital, Palle Juul-Jensens Boulevard 99, 8200 Aarhus, Denmark: senior consultant.<br>2. Department of Clinical Medicine, Aarhus University, Palle Juul-Jensens Blvd. 82, 8200 Aarhus N, Denmark: associate professor.	

Trine Mogensen	1. Department of Infectious Diseases, Aarhus University Hospital, Palle Juul-Jensens Boulevard 99, 8200 Aarhus, Denmark: staff specialist.<br>2. Department of Biomedicine, Aarhus University, Hoegh Guldbergs gade 10, 8000 Aarhus C, Denmark: professor.	0000-0002-1853-9704
William Ullahammer	1. Department of Infectious Diseases, Aarhus University Hospital, Palle Juul-Jensens Boulevard 99, 8200 Aarhus, Denmark:<br>2. Department of Clinical Medicine, Aarhus University, Palle Juul-Jensens Blvd. 82, 8200 Aarhus N, Denmark:	
Won-Yong Kim	1. Department of Cardiology, Aarhus University Hospital, Palle Juul-Jensens Boulevard 99, 8200 Aarhus, Denmark: senior consultant.<br>2. Department of Clinical Medicine, Aarhus University, Palle Juul-Jensens Blvd. 82, 8200 Aarhus N, Denmark: professor.	0000-0003-1302-1671
