# Supplementary material for: Characteristics of a Danish Post-COVID Cohort Referred for Examination due to Persistent Symptoms Six Months after Mild Acute COVID-19
Source: J Clin Med. 2022 Dec 10;11(24):7338. doi: 10.3390/jcm11247338 (PMC9783804; doi:10.3390/jcm11247338)
Supplement: Supplementary file 1 [file jcm-11-07338-s001.zip › Figure S1.pdf]

Symptom Questionnaire

- acute symptoms and post COVID symptoms

Date__________
Name (label)______________________________, Personal id ___________-________

Did you experience symptoms when you first discovered you were infected/first fell ill with COVID-19?

Fill in the date of the first COVID-19 symptom __/__/_____
(fill in the month and year if you do not remember the exact date)

Did you experience the following symptoms	X in 1 of 3 boxes			Duration of symptom in days<br>If you still have the symptom: write "still"	Did have the symptom with the same manifestation before COVID-19
	Yes	No	Don't know		
Headache	☐	☐	☐	____	☐
Dizziness	☐	☐	☐	____	☐
Numbness/tingling/sleeping/sensation of skin (e.g., hands/feet)	☐	☐	☐	____	☐
Impaired smell	☐	☐	☐	____	☐
Impaired taste	☐	☐	☐	____	☐
Confusion/(febrile) delirium	☐	☐	☐	____	☐
Dyspnea	☐	☐	☐	____	☐
Cough	☐	☐	☐	____	☐
Expectoration	☐	☐	☐	____	☐
Chest pain	☐	☐	☐	____	☐
Palpitations	☐	☐	☐	____	☐
Runny nose/nasal obstruction	☐	☐	☐	____	☐
Sore throat	☐	☐	☐	____	☐
Nausea	☐	☐	☐	____	☐
Abdominal pain	☐	☐	☐	____	☐
Diarrhea	☐	☐	☐	____	☐
Skin rash	☐	☐	☐	____	☐
Itching skin	☐	☐	☐	____	☐
Joint pain	☐	☐	☐	____	☐
Swollen joints	☐	☐	☐	____	☐
Myalgia	☐	☐	☐	____	☐
Physical exhaustion	☐	☐	☐	____	☐
Subjective fever	☐	☐	☐	____	☐
Weight loss	☐	☐	☐	_____kg	
Hospitalization	☐	☐	☐	____	

Date___________
Name (label)________________________________, Personal id _____________-________

Symptoms experienced during the last 4 weeks (page 2-3)

	During the past 4 weeks, to what extent have you experienced	X in 1 of 5 boxes					Did experience the symptom with the same manifestation before COVID-19
		None	A little	Some	A lot	Very much	
Central nervous system senses	Headache	☐	☐	☐	☐	☐	☐
	Dizziness	☐	☐	☐	☐	☐	☐
	Numbness/tingling/sleeping/sensation of skin (e.g. hands/feet)	☐	☐	☐	☐	☐	☐
	Impaired smell	☐	☐	☐	☐	☐	☐
	Impaired taste	☐	☐	☐	☐	☐	☐
Cognition	Difficulty concentrating/problems doing what you usually do mentally	☐	☐	☐	☐	☐	☐
	Difficulty remembering things that have just happened	☐	☐	☐	☐	☐	☐
	Difficulty remembering things that happened months or years ago	☐	☐	☐	☐	☐	☐
Heart and lungs	Dyspnea at rest	☐	☐	☐	☐	☐	☐
	Dyspnea at physical activity	☐	☐	☐	☐	☐	☐
	If yes, (dyspnea):<br><br>Tick 1 next to the activity you experience as the worst	1: Are you ever troubled by breathlessness except on strenuous exertion? ☐					
		2: Are you short of breath when hurrying on the level or walking up a slight hill? ☐					
		3: Do you have to walk slower than most people on the level? Do you have to stop after a mile or so (or after ¼ hour) on the level at your own pace? ☐					
		4: Do you have to stop for breath after walking about 100 yds. (or after a few minutes) on the level? ☐					
		5: Are you too breathless to leave the house, or breathless after undressing? ☐					
	Cough	☐	☐	☐	☐	☐	☐
	Expectoration	☐	☐	☐	☐	☐	☐
	Chest pain	☐	☐	☐	☐	☐	☐
	Palpitations	☐	☐	☐	☐	☐	☐
	Runny nose or nasal obstruction	☐	☐	☐	☐	☐	☐

	Sore throat	☐	☐	☐	☐	☐	☐
	During the past 4 weeks, to what extent have you experienced	None	A little	Some	A lot	Very much	Did experience the symptom with the same manifestation before COVID-19
Abdomen	Loss of appetite	☐	☐	☐	☐	☐	☐
	Nausea	☐	☐	☐	☐	☐	☐
	Abdominal pain	☐	☐	☐	☐	☐	☐
	Diarrhea	☐	☐	☐	☐	☐	☐
	Altered bowel habits	☐	☐	☐	☐	☐	☐
	Weight loss after the acute phase of COVID-19	____kg			Weigt loss stopped ☐		
Infection	Fever	Yes☐	No ☐	Dont know ☐	Number of days with feber (past 4 weeks) ____		
	During the past 4 weeks, to what extent have you experienced	None	A little	Some	A lot	Very much	Did experience the symptom with the same manifestation before COVID-19
Skin	Rash	☐	☐	☐	☐	☐	☐
	Itching	☐	☐	☐	☐	☐	☐
Musculoskeletal	Joint pain	☐	☐	☐	☐	☐	☐
	Swollen joints	☐	☐	☐	☐	☐	☐
	Myalgia	☐	☐	☐	☐	☐	☐
	Muscle exhaustion	☐	☐	☐	☐	☐	☐
General symptoms	Physical exhaustion	☐	☐	☐	☐	☐	☐
	Disturbed sleep	☐	☐	☐	☐	☐	☐
	Problems falling asleep	☐	☐	☐	☐	☐	☐
	Awakening	☐	☐	☐	☐	☐	☐

Date__________
Name (label)______________________________, Personal id ____________-________

Other symptoms and worst symptom	
In association with COVID-19, have you had symptoms that are not mentioned on the previous 3 pages?	___________________________________
Symptom/problem that is currently most bothersome after COVID-19 (affects your everyday life the most)	____________________________________

Illness and need to contact hospital/doctor	
Admissions to hospital after the acute phase of COVID-19	Yes ☐ No ☐
If yes	Diagnosis/symptom:____________________________ ____
Contacts to a doctor after the acute phase of COVID-19 (not the contact that gave rise to the current referral)	Yes ☐ No ☐
If yes	Diagnosis/symptom:____________________________ ____

Comments

Date___________
Name (label)_________________________________, Personal id _____________-_________

Work

How is your current work situation do best describe?, or your previous work situation if you are not currently working? (0 or multiple crosses possible)

□ Employed □ Self-employed □ Voluntary work (unpaid)
□ Student □ Stay at home □ Retired □ Newer worked

Which of the following choices best describe your current work situation? (tick 1-2 boxes)

A. If employed, are you then? ☐ Full time ☐ Part time ☐ Partially recovered

B. If not employed, are you then?: ☐ Not working due to your health

☐ Not working due to other reasons: please elaborate:____________________
____

If you are not currently working, what date did you stop? ___ __/__/_____

What is the highest level of education you have completed? (tick 1-2 boxes)
Primary school

□ Not completed primary school □ Primary school □ High school □ Vocational education
□ Medium-cycle higher education □ University □ Master/PhD

What is your current or most recent job/profession (job title)?

____
